# Bactericides Based on Copper Nanoparticles Restrain Growth of Important Plant Pathogens

**DOI:** 10.3390/pathogens9121024

**Published:** 2020-12-05

**Authors:** Adamantia Varympopi, Anastasia Dimopoulou, Ioannis Theologidis, Theodora Karamanidou, Alexandra Kaldeli Kerou, Afroditi Vlachou, Dimitrios Karfaridis, Dimitris Papafotis, Dimitris G. Hatzinikolaou, Alexander Tsouknidas, Nicholas Skandalis

**Affiliations:** 1 Institute of Molecular Biology and Biotechnology, FORTH, 71110 Voutes Heraklion, Greece; avarympopi@biol.uoa.gr (A.V.); anastasia_dimopoulou@imbb.forth.gr (A.D.); ioannis_theologidis@imbb.forth.gr (I.T.); 2 Enzyme and Microbial Biotechnology Unit, Department of Biology, National and Kapodistrian University of Athens, Zografou, 15784 Athens, Greece; jimpa7@gmail.com (D.P.); dhatzini@biol.uoa.gr (D.G.H.); 3 PLiN Nanotechnology S.A., Spectra Business Center 12th km Thessaloniki-Chalkidiki, Thermi, 57001 Thessaloniki, Greece; tk@plin-nanotechnology.com (T.K.); ak@plin-nanotechnology.com (A.K.K.); av@plin-nanotechnology.com (A.V.); 4 Department of Physics, Aristotle University of Thessaloniki, 541 24 Thessaloniki, Greece; dkarfari@physics.auth.gr; 5 Keck School of Medicine of University of Southern California, Health Sciences Campus, 1441 Eastlake Ave, Los Angeles, CA 90033, USA

**Keywords:** copper nanoparticles, particle size, bacterial plant pathogens, susceptibility testing

## Abstract

Copper nanoparticles (CuNPs) can offer an alternative to conventional copper bactericides and possibly slow down the development of bacterial resistance. This will consequently lower the accumulation rate of copper to soil and water and lower the environmental and health burden imposed by copper application. Physical and chemical methods have been reported to synthesize CuNPs but their use as bactericides in plants has been understudied. In this study, two different CuNPs products have been developed, CuNP1 and CuNP2 in two respective concentrations (1500 ppm or 300 ppm). Both products were characterized using Dynamic Light Scattering, Transmission Electron Microscopy, Attenuated Total Reflection measurements, X-ray Photoelectron Spectroscopy, X-ray Diffraction and Scattering, and Laser Doppler Electrophoresis. They were evaluated for their antibacterial efficacy in vitro against the gram-negative species *Agrobacterium tumefaciens*, *Dickeya dadantii*, *Erwinia amylovora*, *Pectobacterium carotovorum*, *Pseudomonas corrugata*, *Pseudomonas savastanoi* pv. *savastanoi*, and *Xanthomonas campestris* pv. *campestris*. Evaluation was based on comparisons with two commercial bactericides: Kocide (copper hydroxide) and Nordox (copper oxide). CuNP1 inhibited the growth of five species, restrained the growth of *P. corrugata,* and had no effect in *X. c.* pv *campestris*. MICs were significantly lower than those of the commercial formulations. CuNP2 inhibited the growth of *E. amylovora* and restrained growth of *P. s.* pv. *savastanoi*. Again, its overall activity was higher compared to commercial formulations. An extensive in vitro evaluation of CuNPs that show higher potential compared to their conventional counterpart is reported for the first time and suggests that synthesis of stable CuNPs can lead to the development of low-cost sustainable commercial products.

## 1. Introduction

Copper-based nanoparticles (CuNPs) have been synthesized since the early 1990s [1,2]. According to the European Commission definition, CuNPs are described as media, where at least 50% of the particles exhibit sizes of below 100 nm [3]. They were initially used for energy conversion and storage, chemical and material manufacturing due to their catalytic (mostly), optical and conductive properties [4,5,6]. Over the past decades, five major methods for Cu-based NPs have been developed including thermal treatment, sonochemical and photochemical techniques, electrochemical synthesis, and chemical treatment [7]. The latter is the most popular among them. Recently, a more ecological, economical, toxic waste-free and efficient method has been produced using biological routes [8]. Now, apart from the methodology, the challenge is to achieve the production of a small range size of CuNPs with strong stability and antimicrobial activity.

Biogenic CuNPs have emerged as a new class of antimicrobials with potential against a range of pathogens [4,9], including those that have developed antibiotic resistance [10,11]. However, the exact mechanism of action of CuNPs is not yet completely understood. One of the most predominant theories is that copper-based NPs damage the cellular membrane by changing its permeability, thus causing cellular death [9,12,13]. Another hypothetical mechanism refers to oxidative cell damage due to protein oxidation, lipid peroxidation and DNA degradation by the overproduction of reactive oxygen species (ROSs) [7,12]. It is also possible that NPs interfere with essential biochemical processes, such as DNA replication by the uptake of their metallic ions [7,12,13]. However, recent studies indicate that the biocidal activity of CuNPs is caused by a combination of different mechanisms, resulting in chemical and physical destruction, while also leading to gene expression inhibition [8,13].

Nano-agriculture is a cutting-edge sector, applied in modern farming, with the potential of significant benefits to food quality, nutrition and safety investigation [14]. Inherently based on reduced chemical inputs, nano-agrochemicals are anticipated to dominate this field shortly, as they reduce the amount of chemicals released into the environment and, thus, have a minimal ecological footprint when compared to conventional pesticides and fertilizers, as several inorganic nanomaterials (NMs), such as copper [15], aluminum [16] and silver [17], have already been recognized for their pesticidal properties. In plant protection, CuNPs have shown efficacy against *Fusarium* sp., *Phoma destructiva*, *Curvularia lunata*, *Alternaria alternata*, *Fusarium oxysporum*, *Penicillium italicum*, *Penicillium digitatum* and, *Rhizoctonia solani* [18]. Moreover, copper nanoparticles of various dimensions (11–14 nm) and chemical forms (Cu_2_O, CuO, and Cu/Cu_2_O) have been reported to control *Phytophthora infestans*, with lesser quantities (15–35 g/hl) than the registered copper-based products (35–224 g/hl) [19]. There are few studies which indicate biocidal effect of CuNPs against bacteria phytopathogens such as *Ralstonia solanacearum*, *Pseudomonas* spp. and *Xanthomonas* spp. [20,21,22] with the vast majority focusing on their effectiveness against human pathogens (*E. coli, P. aeruginosa*, *Klepsiella pneumoniae*, etc.) [7,9,23]. Nanoparticles based on other metals, such as the metallic oxides of Ag, Mg, Si, Zn and carbon have been proved suppressive towards economically important plant bacteria as *Xanthomonas citri* pv. *citri* and *Xylella fastidiosa* [24].

*Agrobacterium tumefaciens* causes considerable economic losses in nurseries while it affects a wide range of plant species [25]. As *A. tumefaciens* is responsible for the crown gall disease, inserts a portion of the tumor-inducing plasmid, transfer DNA, into the plant genome to form tumor galls in plant tissues [26]. *Pseudomonas savastanoi* pv. *savastanoi* which produces olive knots can result in reduced vegetative growth and olive yield and unclear olive oil quality [27]. *Erwinia amylovora*, the causal agent of fire blight, is considered a devastating bacterial pathogen of *Rosaceae* family worldwide [28]. The control of fire blight is challenging due to the absence of synthetic compounds with systemic properties that directly affect the pathogen [29]. *Xanthomonas campestris* pv. *campestris* is a constant concern for cruciferous growers while the disease is particularly devastating in cabbage and kale, where destroyed leaves are rendered unmarketable [30]. *Pseudomonas corrugata*, a ubiquitous phytopathogen, related to Tomato Pit Necrosis (TPN) syndrome, is present in all tomato-growing areas in the world [31]. Another disease with occasionally major losses for solanaceous plants is bacterial stem rot, which is associated with *Pectobacterium carotovorum* (syn. *Erwinia carotovora* pv. *carotovora*) and *Dickeya dadantii* (syn. *Erwinia chrysanthemi*) [32]. However, the current control methods implying the use of chemicals compounds or antibiotics become ineffective due to the natural evolvement of bacterial resistance to these agents [33].

We present here the development of two stable commercial products to be used commercially, which were based on small size copper nanoparticles. The first was based on cupric oxide (CuNP1) and the second on a mixture of cuprous oxide and copper hydroxide (CuNP2). Depending on the composition, e.g., copper oxides or copper hydroxide, the production of CuNPs displays several challenges, such as stability and aggregation. Copper hydroxide, for instance, tends to develop different nanostructures, e.g., nano-wires, -rods, -belts, etc. [34]. Copper hydroxide NPs have also been reported to be metastable, transitioning into copper oxide NPs [35], while their stability is also pH-dependent [36]. In order to synthesize stable CuNPs, of a specific chemical structure, two different stabilizers were used, and the resulting NPs were fully characterized. For the first time, we compare the bactericidal efficacy of CuO NPs with a mixture of Cu_2_O and Cu(OH)_2_ NPs but also with their respective commercial formulation of conventional copper oxide/hydroxide. Comparisons were based on extensive in vitro testing against seven important bacterial phytopathogens [37] of different genera.

## 2. Results

### 2.1. Dynamic Light Scattering

**Figure 1:**
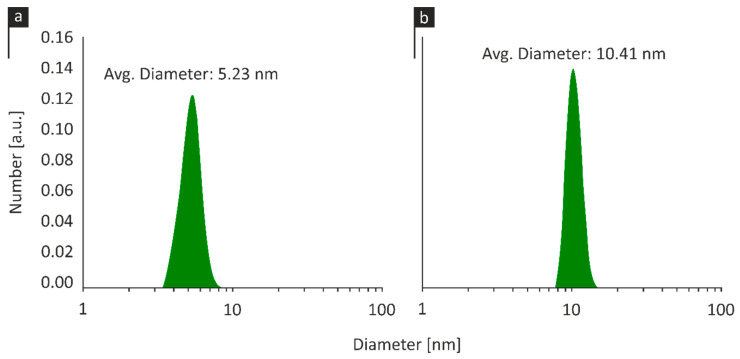
Particle size distribution of (**a**) copper-based nanoparticle (CuNP) 1 and (**b**) CuNP2.

The size distribution of CuNP1 as depicted in Figure 1a, indicating the presence of a monodisperse population of nanoparticles with an average size of 5.23 ± 0.8 nm. Similarly, CuNP2 presented a narrow size distribution with an average particle size of 10.41 ± 1.2 nm (Figure 1b), being evident of larger size values compared to CuNP1.

### 2.2. Transmission Electron Microscopy

**Figure 2:**
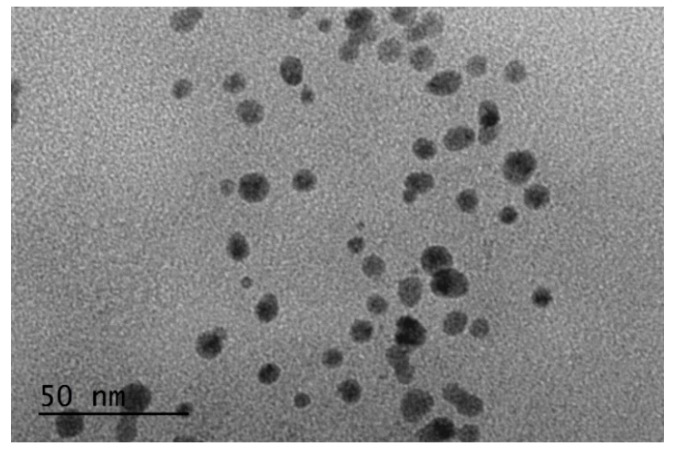
Transmission electron microscopy (TEM) image of CuNP1.

High-Resolution Transmission Electron Microscopy (HR-TEM) images of the CuNPs indicated that both species were of a spherical morphology with an average size between 5 and 10 nm. An HR-TEM image of CuNP1 is indicatively depicted in Figure 2.

### 2.3. Attenuated Total Reflection Measurements

**Figure 3:**
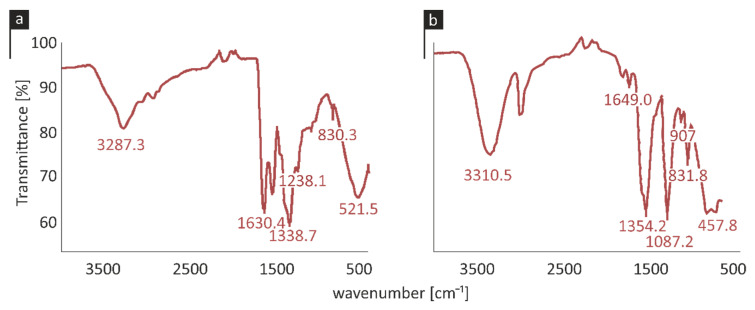
Attenuated total reflection (ATR) spectra of (**a**) CuNP1 and (**b**) CuNP2.

Attenuated total reflection (ATR) was employed to analyze the stretching and bending frequencies of the molecular functional groups of the prevalent copper species and of each stabilizer. The ATR spectrum of CuNP1 is shown in Figure 3a. The CuNP1 ATR spectrum presented the characteristic peaks of the stabilizer S1 at 3287, 1630, 1539 and 1238 cm^−1^. The transmittance peak at 1630 cm^−1^ also indicates a symmetrical stretching vibration of the Cu-O bond, while the characteristic peak at 521 cm^−1^ corresponds to a bending vibration of the Cu-O bond [38,39,40].

CuNP2 presented an absorption peak at 605 cm^−1^, indicating a Cu(I)-O vibration, which could be attributed to the presence of cuprous oxide species [41]. The ATR spectrum also showed an absorption peak at 1354 cm^−1^, corresponding to a Cu-OH bond and a peak at 907 cm^−1^ indicating a Cu-OH vibration. The peak at 475 cm^−1^ represented a characteristic peak for Cu(OH)_2_ [40]. The main peaks of S2 were also observed at 3310, 2921, 1649, 1354, 1087 and 831 cm^−1^, as illustrated in Figure 3b.

### 2.4. X-Ray Photoelectron Spectroscopy

XPS data revealed that the prevalent copper species in CuNP1 was cupric oxide (CuO), whereas CuNP2 was determined to host both cuprous oxide (Cu_2_O) and copper hydroxide (Cu(OH)_2_) in 64% and 36% ratio, respectively.

### 2.5. X-Ray Diffraction and Scattering

XRD data confirmed the existence of one type of copper-based nanoparticles in CuNP1, as well as the presence of two species in CuNP2.

### 2.6. Laser Doppler Electrophoresis

The zeta potential of copper-based nanoparticles was measured, in order to evaluate the surface charge of nanoparticles in an aqueous solution. CuNP1 presented a surface charge at −12.23 ± 0.9 mV, while CuNP2 at −4.64 ± 0.4 mV. The physicochemical characteristics of CuNPs are presented in Table 1.

### 2.7. Susceptibility Testing of CuNPs against Bacterial Phytopathogens

**Figure 4:**
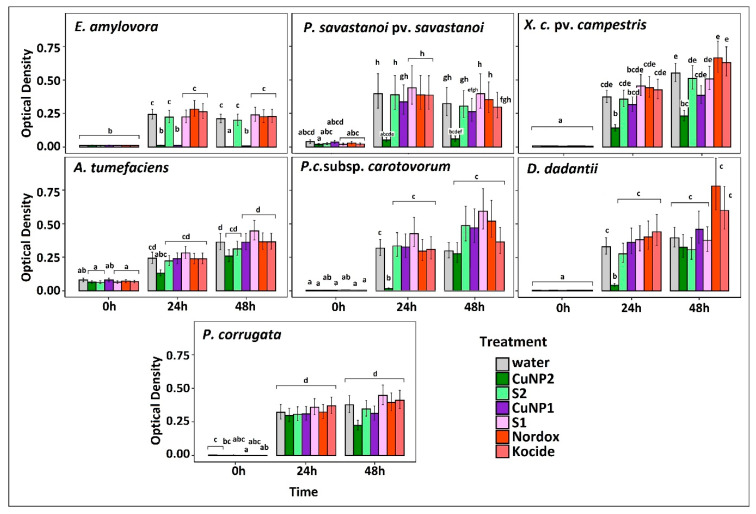
Susceptibility testing of bacterial pathogens based on growth in the presence of 240 ppm CuNP1 and CuNP2. Their respective stabilizers (S1 and S2) and water treatment were used as controls, while Nordox and Kocide were used as reference compounds at 240 ppm. The effect is evaluated in OD600 measured at 0, 24, and 48 h post inoculation (hpi) using a multi-detection microplate reader. Estimated marginal means and their standard errors for three independent experiments of triplicate data sets are plotted here. Different letters (a–h) represent statistically different data points at *p* ≤ 0.05 according to Tukey post hoc comparisons.

CuNP1 and CuNP2, the two NP products that were described above, had the final copper concertation of 1500 and 300 ppm, with the maximum applicable concentration for in vitro experiments reaching 1200 and 240 ppm, respectively. Eight economically important plant pathogenic bacteria were tested against a 240 ppm concentration of CuNP1 (purple) and CuNP2 (green) for susceptibility testing. Their respective stabilizers (S1 and S2) and water treatment were used as controls. Nordox and Kocide, commercial formulations of copper oxide and copper hydroxide, were used as reference compounds at an equal concentration (Figure 4).

In the case of CuNP1, growth was inhibited only in the case of *E. amylovora* which difference was significant compared to controls. It was marginally (non-significantly) delayed in the case of *P. savastanoi* and had no significant effect in all other cases. All species were further tested against higher CuNP1 concentrations, which were scaled up to 1200 ppm.

CuNP2 inhibited the growth of *E. amylovora* and *P. savastanoi* and difference were statistically significant compared to S1 and water treatments (Figure 4). Interestingly, it was significantly more effective compare to the reference treatments (24 and 48 h post inoculation; hpi). It also restrained significantly the growth of *X.c.* pv *campestris*. These three species were selected for further testing (dose–response). CuNP2 delayed growth (24 hpi) of *A. tumefaciens*, *P.c.* subsp. *carotovorum* and *D. dadantii* compared to controls. Again, it was significantly more effective compared to reference treatments at 24 h. It had no significant effect against *P. corrugata* and neither did the reference treatments. Based on synthesis methodology, CuNP2 could not be scaled up for dose–response experiments.

#### Dose–Response Effect of CuNPs against Bacterial Phytopathogens

**Figure 5:**
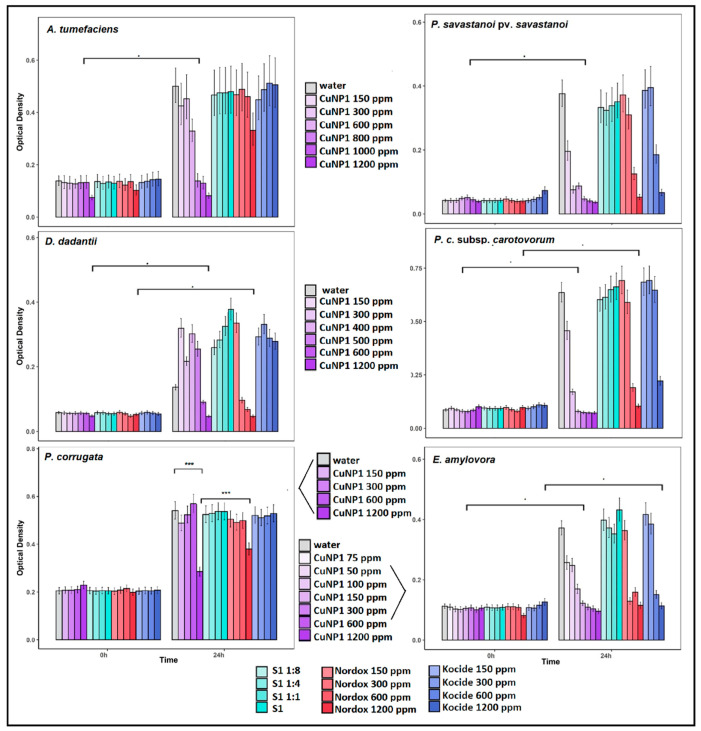
Dose–response effect of CuNP1 (purple) increasing concentrations against three bacterial pathogens. Stabilizer S1 and water treatment were used as controls, while Nordox and Kocide were used as reference compounds. Effect is evaluated in OD 600 at 0 and 24 hpi using methods described in Figure 4. * : *p* ≤ 0.01, *** : *p* = 0 according to Tukey post hoc comparisons.

**Figure 6:**
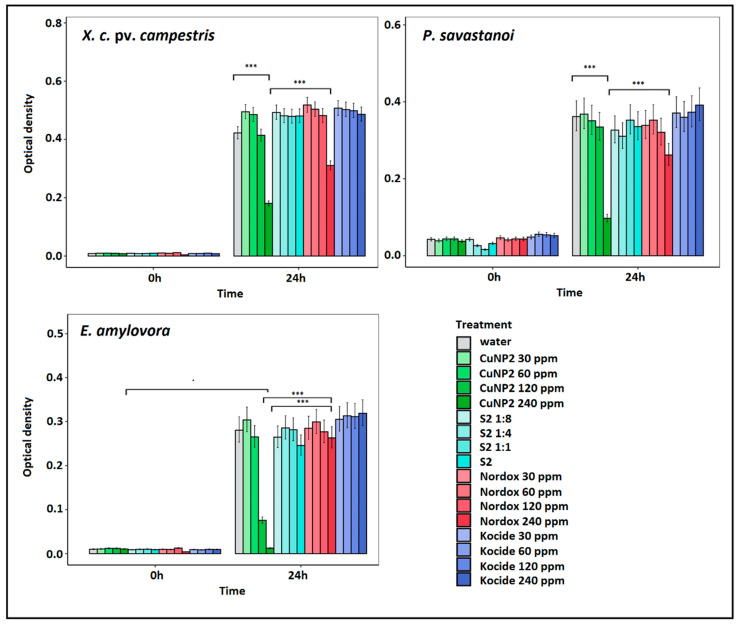
Dose–response effect of CuNP2 (green) concentrations against three bacterial pathogens. Stabilizer S2 and water treatment were used as controls, while Nordox and Kocide were used as reference compounds. Effect is evaluated in OD 600 at 0 and 24 hpi using methods described in Figure 4. * : *p* ≤ 0.01, *** : *p* = 0 according to Tukey post hoc comparisons.

Pathogens that were found sensitive during the first screening (Figure 4) were submitting to an extensive assessment against increasing concentration of both types of nanoparticles for 48 h. The full results are depicted in Figure S1, while the results of the first 24 h are depicted here (Figure 5 and Figure 6) for simplicity and to avoid confusion, because non-susceptible bacterial cell cultures enter the death phase after 24 h. MIC calculations at 24 hpi are shown in Table 2.

**Figure 7:**
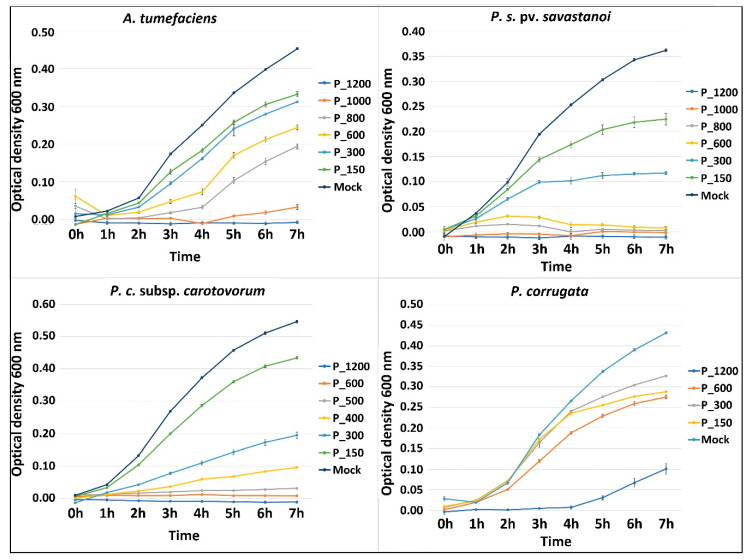
Growth kinetics of selected species in increasing concentrations of CuNP1. OD was measured for 7 h on an hourly basis in microtiter plates incubated at 28 °C. Averages of three replicates in a single experiment are shown here.

CuNP1 was found to be more bactericidal in comparison with respective concentrations of Nordox and Kocide in the case of most pathogenic strains (Table 2; Figure 5). In particular, MIC of *≤*800 ppm was calculated in the case of CuNP1 but not of Nordox and Kocide in *A. tumefaciens* cultures. Growth kinetics showed that concentrations as low as 600 ppm delay significantly bacterial growth (Figure 7). MIC was lower for CuNP1 than for commercial formulations in the case of *P. savastanoi* pv. *savastanoi* (≤800 compared to ≤1200 ppm) and *P. c.* subsp. *carotovorum* (≤400 compared to ≤1200 ppm). Similarly, in the case of *E. amylovora* MIC of CuNP1 was of ≤150 ppm compared to MIC of Kocide that was of ≤1200 ppm while no susceptibility was found in the case of Nordox. Growth kinetics confirmed that concentrations as low as 150 ppm or 300 ppm (for each pathogen, respectively) significantly delayed growth (Figure 7). *D. dadantii* was more susceptible to CuNP1 compared to Kocide but less compared to Nordox (MICs of ≤1200, N/A, or ≤1200 ppm, respectively). *P. corrugata* was confirmed to be of reduced susceptibility; only 1200 ppm of CuNP1 significantly delayed growth (Figure 7) and allowed for MIC50 to be calculated.

The increased susceptibility of *E. amylovora* against CuNP2 was confirmed in dose–response experiments (Figure 6). MIC, MIC90 and MIC50 of ≤240, 96, and 210 ppm were calculated (Table 2). MIC50 was of 210 in the case of *X. c.* pv. *campestris* or 200 ppm in the case of *P. savastanoi* pv. *savastanoi*. In all three cases, bacterial species were more susceptible to CuNP2 compared to reference treatments (Nordox and Kocide).

In the case of susceptible bacterial species, MICs coincided with Minimum Bactericidal Concentration (MBC) (Table 2), suggesting a bactericidal effect.

## 3. Discussion

Nowadays, the crop protection market faces three major challenges. First, the ongoing problem of emergence and transmission of bacterial resistance [33,42]. Second, the reduction of registered pesticides due to residual effects and environmental hazards [43]. Third, there is an increasing awareness among consumers who prefer food products produced in more environmentally friendly ways [44]. Hence, it becomes apparent that alternate, plant protection approaches are inevitable.

Synthesis of CuNP in this work leads to NP of narrow size distribution, suggesting that our production method results in a reliable product with repetitive bactericidal effect. CuNPs had a smaller size compared to others that have been previously reported [8,45,46]. CuNP1 contained 100% CuO and was half the size of CuNP2, which contained 64% Cu_2_O and 36% Cu(OH)_2_.

Results indicated that NPs had an antimicrobial effect against all tested pathogens. Growth inhibition or bactericidal action was enhanced in comparison to commercial copper formulations, Nordox and Kocide. Susceptibility was dependent on the type of NPs rather than the genus of targeted species. In specific, CuNP1 showed higher activity and wider target range compared to CuNP2. Based on previous reports which favored Cu_2_O activity compared to that of CuO [47] and the fact that bacterial sensitivity was higher in the case of conventional copper oxide than copper hydroxide in our experiment, we conclude that synthesis of pure copper oxides of the lowest possible size and higher negative charge increases their activity.

CuNPs had no phytotoxicity effect in plants sprayed with concentrations equal or higher to those recommended (MIC) (Supplement Figure S2). Nano Copper compounds tested in this study can be synthesized in large quantities and at a cost similar to that of conventional copper pesticides. Considering that effective dosage of CuNPs can be eight times lower than that of conventional copper formulations (CuNP1 MIC ≤ 150, Kocide MIC ≤ 1200), copper nanoparticles could offer an environmentally friendly alternative to conventional pesticides [24], at a market discount. Recent studies have shown that Cu-based NPs have an enhanced antiparasitic action when compared to conventional Cu-based chemicals, as they utilize only trace amounts of Cu and thus are less harmful to the plant [24,48]. This also leads to significantly lower (if any) copper accumulation in the environment. Nevertheless, NPs tend to aggregate in their suspension media, e.g., during storage. This is widely accepted to occur within days of production and there is additional literature concerning Cu-based NPs metastability, i.e., Cu NPs tend to transit from one species to another Cu hydroxide to Cu oxide [35]. In contrast to this, the colloidal solutions investigated here are capable of maintaining their compositional integrity for prolonged times, exceeding several months and are thus considered to maintain their efficacy.

In this work, two different types of CuNPs were developed. Both showed superior bactericidal activity in comparison to their respective commercial copper compounds. It was also shown that the size and type of copper oxide determine their bactericidal activity of nanoparticles. Commercial production of such stable and pure compounds will enforce the currently depleted arsenal of bactericidal pesticides.

## 4. Materials and Methods

### 4.1. Nanoparticle Materials

Copper (II) nitrate hemi-pentahydrate (>98% Cu(ΝO_3_)_2_·2.5H_2_O, Mr = 232.59 g/mol) was used as copper precursor and purchased from Alfa Aesar, while sodium hydroxide (>98% NaOH) was purchased from CHEM-LAB. Two stabilizers were used for the synthesis of two different copper-based nanoparticles. Stabilizer 1 (S1) refers to an animal protein with a molecular mass between 20,000 and 25,000 g/mol and PI at 4.7–5.4 and was purchased from Sigma Aldrich. Stabilizer 2 (S2, purity 98–99%) refers to a non-ionic polymer with a molecular mass of 57,000–66,000 g/mol and was purchased from Alfa Aesar. All reagents were used as received, without any further purification.

### 4.2. Synthesis of Copper-Based Nanoparticles

Copper-based nanoparticles were synthesized, modifying wet chemistry approaches to achieve higher productivity and repeatability [11,40,49]. Copper (II) nitrate hemi-(pentahydrate) was selected as the precursor salt and sodium hydroxide (NaOH) as the coordination and pH-adjusting agent. In addition, two different stabilizing agents, namely S1 and S2, were utilized for the synthesis of copper-based nanostructures.

#### 4.2.1. Synthesis of Copper-Based Nanoparticles Using the Protein-Based Stabilizer S1

Copper salt and stabilizer S1 were dissolved separately in deionized water. The copper salt solution was magnetically stirred for 15 min to ensure complete dissolution. The pH of the stabilizer solution was adjusted to pH = 10–11, using 0.5M NaOH prior to the synthesis to avoid precipitation of the protein-based S1 (PI 4.7–5.4). The copper salt solution was then added dropwise to the solution of the S1 under stirring at ambient conditions, while also retaining the pH in the range of 9–11. The color of the solution changed to purple, thus indicating the formation of CuNPs.

#### 4.2.2. Synthesis of Copper-Based Nanoparticles Using the Polymer-Based Stabilizer S2

Similarly, copper salt and stabilizer S2 were separately dissolved in deionized water. The stabilizer solution was rapidly added to the copper solution and 0.5M NaOH was added dropwise under magnetic stirring at ambient conditions, up to the point where the pH was in the range of 10–11.

As soon as the NaOH was added to the copper salt, the color of the solution turned into bright green, thus indicating the formation of CuNPs.

### 4.3. Physicochemical Characterization

The physicochemical features of both CuNPs solutions were evaluated, using several analytical techniques. Particle size and distribution profiles were obtained by Dynamic Light Scattering (DLS), using a VASCO 3 DLS analyzer of Cordouan Technologies. High-Resolution Transmission Electron Microscopy (HR-TEM) was employed to verify the size of the CuNPs, while providing information on their morphology and shape (JEOL JEM 2010 & Oxford INCA). Attenuated Total reflectance (ATR) was used to analyze the resulted copper-based nanoparticles, using a Cary 630 FTIR Spectrometer by Agilent Technologies with a Diamond ATR sampling accessory, while X-ray Photoelectron Spectroscopy (XPS) was employed to determinate and quantify the prevalent copper species, using an AXIS Ultra^DLD^ system by Kratos Analytical (Shimadzu Group Company). Compositional characteristics were validated through X-ray Diffraction and Scattering (XRD), performed on a Bruker D8 ADVANCE device. Finally, a Laser Doppler Electrophoresis (LDE) technique was used to measure the zeta-potential of copper nanoparticles, by using a Wallis Zeta analyzer, Cordouan.

### 4.4. Bacterial Strains and Growth Conditions

Bacterial strains included *Agrobacterium tumefaciens* 1784 BPIC, *Dickeya dadantii* EchPT1 BPIC, *Erwinia amylovora* 842 BPIC, *Pectobacterium carotovorum* subsp. *carotovorum* isolate 3412/17 BPIC, *Pseudomonas corrugata* 870 BPIC, *Pseudomonas savastanoi* pv. savastanoi 1784 BPIC and *Xanthomonas campestris* pv. *campestris* 1656 BPIC. Strains were obtained from the Benaki Phytopathological Institute Collection (BPIC) (http://www.wfcc.info/ccinfo/collection/by_id/610). They were routinely grown at 28 °C in Luria-Bertani (LB) broth or on LB agar medium. LB growth was optimized for all bacterial species so that max growth levels did not differ among them. In specific measurements of cfu/mL of 24 h cultures of *P. savastanoi*, *P. c. subsp. carotovorum*, *E. amylovora* and *A. tumefaciens* after serial dilutions and plating in NA medium, gave populations of 3 × 10^8^, 3 × 10^8^, 1 × 10^8^ and 2 × 10^8^, respectively. These data are in accordance with the literature where Pseudomonads are grown optimally in LB medium [50,51].

### 4.5. Broth Microdilution Method

Every bacterial strain was grown exponentially in overnight cultures and was streaked on selective growth media, respectively, checking bacterial cells appearance (color, pigment and shape), to ensure culture purity. Culture aliquots were then adjusted to a final concentration of 5 × 10^6^ cfu/mL. The implemented method has been described by Skandalis et al. [23], with the following alterations: One target bacterium was assayed on each microtiter plate (Greiner CELLSTAR^®^ 96 well microplates F-bottom) in a single CuNP product (successive serial dilutions), its stabilizer and the two-reference copper-based bactericides Kocide and Nordox in the same concentrations, respectively, and control as well, representing one experiment, in triplicates. Blank wells (treatments and medium only) containing each test concentration were also included in duplicates. Three independent biological experiments were performed for each bacterium. Plates were incubated in ZWR-240 Incubator Shaker (Labwit Scientific Pty Ltd., Burwood East, Victoria, Australia), at 28 °C and 200 rpm for 48 h. OD absorbance (600 nm) was measured at 0, 24 and 48 h after inoculation in triplicate readings with a 25-s shake by a microplate monochromator-based UV/VIS spectrophotometer (Multiskan GO, Thermo Fisher Scientific Corporation: Waltham, MA, USA). The effect of CuNPs on bacterial growth was assessed by determining Minimum Inhibitory Concentration (MIC), MIC90, MIC50, and Minimum Bactericidal Concentration (MBC) [23]. MBC was determined from broth microdilution tests by subculturing to agar plates that do not contain the test agent. MBC is identified by determining the lowest concentration of antibacterial agent that reduces the viability of the initial bacterial inoculum by ≥99.9%.

### 4.6. Data Analysis

Measurements for each species were collected in triplicated 96-well plates, which were considered as different experimental blocks. OD measurements were modeled using Linear Mixed Effects Models (LMMs). Blank estimates of each treatment combination were subtracted from the corresponding experimental ODs, a fact that reduced initial values towards zero. Estimates (MIC, MIC90, MIC50) were calculated in statistical language as discussed in Skandalis et al. [23]. Estimated marginal means were retrieved with the emmeans function of the emmeans package for each combination of time and concentration.

## Figures and Tables

**Table 1:** Physicochemical characteristics of copper-based nanoparticles.

	CuNPs-S1	CuNPs-S2
pH	10.0–10.5	10.0–10.5
Size (nm)	5.23 ± 0.8	10.41 ± 1.2
Zeta-potential (mV)	−12.23 ± 0.9	−4.64 ± 0.4
Copper Species	CuO (100%)	Cu_2_O (64%), Cu(OH)_2_ (36%)

**Table 2:** Calculated MICs and MBCs of broth efficacy tests of CuNPs as depicted in Figure 5 and Figure 7.

	CuNP1 (Purple-S1) ppm				CuPN2 (Green-S2) ppm				Nordox ppm	Kocide ppm
Species	MIC	MIC_50_	MIC_90_	MBC	MIC	MIC_50_	MIC_90_	MBC	MIC	MIC
*Agrobacterium tumefaciens*	800	670	790	800						
*Dickeya dadantii*	1200	540	600	1200					1200	-
*Erwinia amylovora*	150	110	-	150	240	96	210	240	-	1200
*Pectobacterium carotovorum subsp. carotovorum*	400	250	230	400					1200	-
*Pseudomonas corrugata*	-	1000	-							
*Pseudomonas savastanoi pv. savastanoi*	800	250	720	800	-	200	-		1200	1200
*Xanthomonas campestris pv. campestris*					-	210	-			

## Supplementary Materials

The following are available online at https://www.mdpi.com/2076-0817/9/12/1024/s1, Figure S1: Dose–response effect of CuNP1 and CuNP2 against bacterial pathogens at a 48 h period. Figure S2: 6-week-old tomato plants, 2 days after being sprayed with CuNP1, water (Mock) and CuNP2.
