# Supplementary material for: Bactericides Based on Copper Nanoparticles Restrain Growth of Important Plant Pathogens
Source: Pathogens. 2020 Dec 5;9(12):1024. doi: 10.3390/pathogens9121024 (PMC7762092; doi:10.3390/pathogens9121024)
Supplement: Supplementary file 1 [file pathogens-09-01024-s001.pdf]

pathogens

MDPI

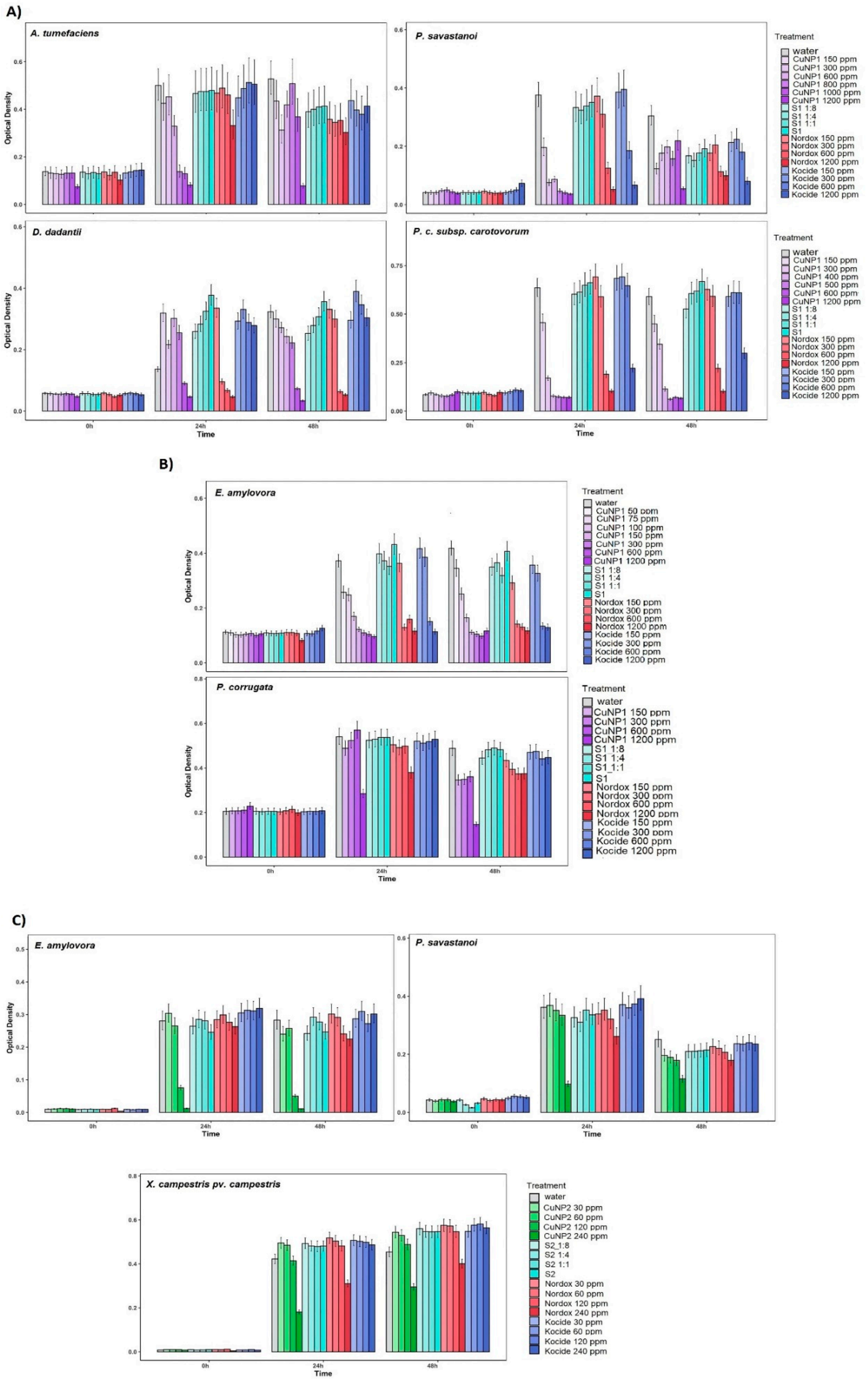

Supplement Figure S1. Dose-response effect of CuNP1 (A and B) and CuNP2 (C) concentrations against various bacterial pathogens. Effect is evaluated in OD600 at 0, 24 and 48 hpi of microtiter plates incubated at 28 °C. Estimated marginal means and their standard errors calculated by LMMs (see methods) for three independent experiments of triplicate data sets are plotted here.

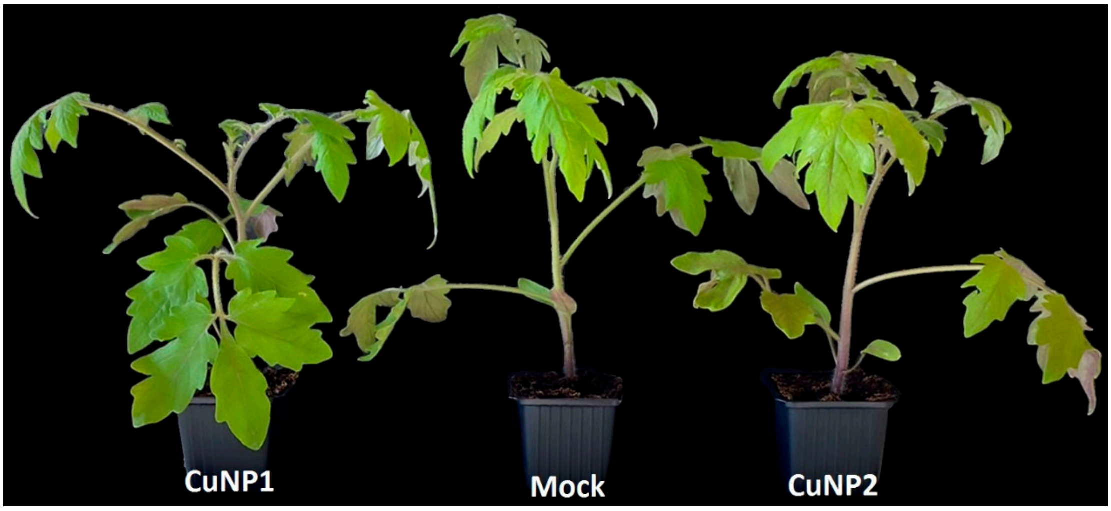

Supplement Figure S2. 6-week-old tomato plants, 2 days after sprayed with water (Mock) or the high concentration of CuNP1 (1500 ppm) and CuNP2 (300 ppm), which exceeded those tested in *in vitro* experiments of bacterial pathogens (1200 ppm and 240 ppm for CuNP1 and CuNP2, respectively).
